# Dataset of whole-brain resting-state fMRI of 227 young and elderly adults acquired at 3T

**DOI:** 10.1016/j.dib.2021.107333

**Published:** 2021-08-28

**Authors:** Xia Li, Håkan Fischer, Amirhossein Manzouri, Kristoffer N.T. Månsson, Tie-Qiang Li

**Affiliations:** a Institute of Informatics Engineering, China Jiliang University, Hangzhou, China; b Department of Psychology, Stockholm University, Sweden; c Stockholm University Brain Imaging Centre, Sweden; d Department of Clinical Science, Intervention, and Technology, Karolinska Institute, Sweden; e Department of Medical Radiation and Nuclear Medicine, Karolinska University Hospital, Sweden

**Keywords:** Quantitative data-driven analysis (QDA), Resting-state functional magnetic resonance imaging (R-fMRI), Resting-state functional connectivity (RFC), Connectivity strength index (CSI), Connectivity density index (CDI), Adult age

## Abstract

To investigate the impact of adult age on the brain functional connectivity, whole-brain resting-state functional magnetic resonance imaging (R-fMRI) data were acquired on a 3T clinical MRI scanner in a cohort of 227, right-handed, native Swedish-speaking, healthy adult volunteers (N=227, aged 18-74 years old, male/female=99/128). The dataset is mainly consisted of a younger (18-30 years old n=124, males/females=51/73) and elderly adult (n=76, 60-76 years old, males/females=35/41) subgroups. The dataset was analyzed using a new data-driven analysis (QDA) framework. With QDA two types of threshold-free voxel-wise resting-state functional connectivity (RFC) metrics were derived: the connectivity strength index (CSI) and connectivity density index (CDI), which can be utilized to assess the brain changes in functional connectivity associated with adult age. The dataset can also be useful as a reference to identify abnormal changes in brain functional connectivity resulted from neurodegenerative or neuropsychiatric disorders.

## Specifications Table

**Table:** 

Subject	Biological sciences Neuroscience
Specific subject area	Brain Imaging
Type of data	BOLD fMRI data of human brain, estimates of functional connectivity netrucs and impact of adult age
How data were acquired	Resting-state functional MRI on a whole-body 3T clinical MRI scanner (Magnetom Trio, Siemens Medical Solutions)
Data format	Raw and processed
Parameters for data collection	A time series of 150 dynamic timeframes of volumetric images with matrix size=76 × 76 × 34. TE/TR 35/2500 ms, 34 slices of 3.5 mm thick, and FOV = 225 mm.
Description of data collection	The MRI data acquisition was conducted on a whole-body 3T clinical MRI scanner (Magnetom Trio, Siemens Medical Solutions, Erlangen, Germany) equipped with a 32-channel phased-array receiving head coil. All data was acquired at Karolinska University Hospital, Huddinge, Stockholm, between noon and 5:00 PM. The MRI data acquisition protocol included the following scanning sessions: (1) 3-plane localizer to guide remaining data sampling; (2) Conventional clinical MRI scans including 3D T1-weighted MPRAGE, T2 and FLAIR scans; (3) A session of 375 s long R-fMRI measurements. The main acquisition parameters for the R-fMRI data included the following: TE/TR 35/2500 ms, flip angle = 90°, 34 slices of 3.5 mm thick, FOV = 225 mm, matrix size = 76 × 76, data acquisition acceleration with GRAPPA parallel imaging method (iPAT = 2), and 150 dynamic timeframes.
Data source location	Institution: Karolinska Institute City/Town/Region: Stockholm Country: Sweden Primary data sources: DICOM format
Data accessibility	Li, Tie-Qiang (2021), “resting-state fMRI data for Normal adults”, Mendeley Data, V1, http://dx.doi.org/10.17632/pt9d2rdv46.1 http://dx.doi.org/10.17632/pt9d2rdv46.1 Li, Tie-Qiang (2021), “QDA-processed”, Mendeley Data, V1, http://dx.doi.org/10.17632/3nm3ctwycc.1 http://dx.doi.org/10.17632/3nm3ctwycc.1
Related research article	Not available

## Value of the Data

• The dataset can be used to assess brain functional connectivity changes associated with adult age. It can also be used as reference to detect abnormal changes associated with neuropsychiatric disorders.
• Neural scientists who study brain functions and aging of the brain may be beneficial. Physicians of neurological disciplines can use the dataset as reference to assess abnormal brain functional changes in patients. Data scientists who are interested in developing frameworks to assess normal and abnormal brain functional connectivity can use the dataset to train or test their frameworks.
• The dataset can add to existing R-fMRI database of similar acquisition conditions. It can also be used to assess how the brain functional connectivity metrics are influenced by data acquisition parameters, when compared with datasets of different acquisition parameters.

## 1 Data Description

The raw data link at http://dx.doi.org/10.17632/pt9d2rdv46.1 is a compressed tar file for the folder named as raw_fmri_2. The raw data file folder contains 227 compressed 16-bit binary NIFTI files, one for each participant. The 1st alphabet in the file names indicates the gender of the volunteers: “f” for female and “m” for male. The 2nd and 3rd digits in the file names indicate the age of volunteers. The remaining alphabets and numerical digits of the file names are randomized to encode the individual subject. These files are for the resting-state fMRI raw data organized into a time series of 3D volumes. The basic information for the raw data files is provided in Table 1.

**Table 1:** Summary of R-fMRI raw data file information.

Direction	FOV	Resolution	Voxel Size
R-to-L	225mm	3mm	76
A-to-P	225mm	3mm	76
I-to-S	133mm	3.5mm	39
Time	≥150 frames	2.5s	≥150

R-to-L: right to left; A-to-P: anterior to posterior; I-to-S: inferior to superior.

The raw data link at http://dx.doi.org/10.17632/3nm3ctwycc.1 contains the processed data files. It includes the computed QDA functional connectivity metrics for all the subjects (see Table 2), the preprocessing shell script (preproc4QDA_4mmke4) and a folder with the auxiliary brain template images. The processed connectivity metrics data files are provided in a 4D-nifti format (16-bit binary) obtained by concatenating individual's 3D-volumetric QDA metric in order of the R-fMRI raw data file list. Since the QDA metrics were computed in the MNI152 standard template space (https://fsl.fmrib.ox.ac.uk/fsl/fslwiki/Atlases) at 4mm isotropic spatial resolution, the matrix size of the QDA metric data files is 45 × 54 × 45 corresponding to the FOV of 180 × 216 × 189 mm^3^. The preprocessing shell script is in ASCII text format and requires the AFNI software package (https://afni.nimh.nih.gov/) and Linex system. The preprocessing of the R-fMRI raw data can be automatically completed by executing the shell script in the directory containing all the R-fMRI raw data files. The MNI152 templates of different resolutions were given in the folder for auxiliary data files.

**Table 2:** Summary of the processed QDA functional connectivity metric data files.

File name	Matrix Size	Data information
Z_CSI.nii.gz	45 × 54 × 45 × 227	Z-score of the CSI metrics
Z_CSI_P.nii.gz	45 × 54 × 45 × 227	Z-score of the CSI_P_ metrics
Z_CSI_N.nii.gz	45 × 54 × 45 × 227	Z-score of the CSI_N_ metrics
Z_CDI_k4.nii.gz	45 × 54 × 45 × 227	Z-score of the CDI metrics for kernel k_4_
Z_CDI_k4_P.nii.gz	45 × 54 × 45 × 227	Z-score of the CDI_P_ metrics for kernel k_4_
Z_CDI_k4_N.nii.gz	45 × 54 × 45 × 227	Z-score of the CDI_N_ metrics for kernel k_4_

## 2 Experimental Design, Materials and Methods

### 2.1 Experimental design

The study was aimed to investigate the impact of adult age on the resting-state functional connectivity (RFC) in the brains of healthy adults in a threshold-free and model-free fashion. Therefore, we collected whole-brain resting-state functional magnetic resonance imaging (R-fMRI) data on a whole-body 3T clinical MRI scanner from a cohort of normal adult volunteers. Furthermore, we developed a quantitative data-driven analysis (QDA) method to compute threshold-free voxel-wise RFC metrics. The experimental design to collected R-fMRI raw data and derive RFC metrics involved the following aspects: 1) Recruitment of the study subjects and acquisition of R-fMRI time-series raw data; 2) Preprocessing pipeline of the R-fMRI data; 3) Computation of the threshold free RFC metrics.

For comparison the subject recruitment was mainly consisted of a younger (18-30 years old n=124, males/females=51/73) and elderly adult (n=76, 60-76 years old, males/females =35/41) subgroups. To reduce possible confounding factors, the participants must fulfill certain basic demographic and neuropsychiatric criteria. They must be right-handed, native Swedish speaking, no history of neurological, psychiatric, and cardiovascular disease, and no remarkable neuroanatomical abnormality. All the MRI data were acquired from the same 3T MRI scanner with the same hardware and software settings. The R-fMRI data were acquired by leveraging a multi-slice 2D gradient recalled echo (GRE) echo-planar imaging (EPI) protocol which is sensitive to the blood oxygen level dependent (BOLD) contrast. The acquisition parameters were designed to achieve the optimal contrast-to-noise ratio for the BOLD signal fluctuations based on the hardware limitations. An up-to-date preprocessing pipeline was adopted to compute the voxel-wise correlation coefficient (CC) matrix from each R-fMRI time-series raw data. The QDA framework was designed to derive two types of threshold-free voxel-wise RFC metrics: namely the connectivity strength index (CSI) and connectivity density index (CDI), which can be further statistically tested to assess the brain functional changes associated with adult age. Further details of the different aspects are described below.

### 2.2 Materials

A total of 227 volunteers (aged 18-76 years old, male/female=99/128) completed the study and were recruited into the study through the local social media advertisement in the Stockholm region. All participants were tested to be right-handed, and native Swedish speakers with normal or corrected-to-normal vision. They are all free of a history of neurological, psychiatric and cardiovascular diseases through questionnaire screening and medical examinations. None of the participants reported any use of psychotropic drugs. All participants signed informed consent before completing the MRI examination protocol. They were financially compensated for their participation.

### 2.3 MRI data acquisition procedures

The MRI data acquisition was performed on a whole-body 3T clinical MRI scanner (Magnetom Trio, Siemens Medical Solutions, Erlangen, Germany) equipped with a 32-channel phased-array receiving head coil. All data was acquired at Karolinska University Hospital, Huddinge, Stockholm. The acquisition protocol included the following main scanning sessions:

1) 3-plane localizer.
2) Conventional anatomical MRI scans including 3D T1-weighted MPRAGE, T2 and FLAIR scans. The T_1_-weighted MPRAGE images used for co-registration with functional images were acquired with the following parameters: TR = 1900 ms, TE = 2.52 ms, FA = 9 degrees, FOV = 256, voxel size 1 × 1 × 1 mm. The acquisition parameters for the FLAIR image were the following: TE/TR=89/9000 ms, flip angle=130°; inversion time TI = 2500 ms, slice thickness = 4 mm, FOV=199 × 220 mm.
3) A session of 375 s long R-fMRI measurements using a 2D GRE-EPI pulse sequence emphasizing T_2_*-contrast. The main acquisition parameters for the R-fMRI data acquisition included the following main parameters: TE/TR 35/2500 ms, flip angle = 90°, 39 axial slices of 3.5 mm thick, FOV = 225 mm, matrix size = 76 × 76, sampling bandwidth of 2790 Hz/pixel, and 150 dynamic timeframes. To reduce the data sampling time and mitigate susceptibility-related artifacts, acquisition acceleration with GRAPPA parallel imaging method was used to gain an acceleration factor of 2 along the phase encoding direction. An experienced neuroradiologist inspected the structural anatomic image for all the participants and remarkable pathological findings were detected.

### 2.4 Preprocessing pipeline

The R-fMRI datasets underwent a preprocessing procedure [1,2], which was performed with AFNI (Version Debian-16.2.07∼dfsg.1-3∼nd14.04+1, http://afni.nimh.nih.gov/afni) programs with a bash shell wrapper named as *preproc4QDA_4mmk4*. The preprocessing pipeline included the following steps:

1) Temporal de-spiking of the R-fMRI time series data using the 3dDespike program.
2) Image registration based on six-parameter rigid body motion algorithm was performed to correct for involuntary head motions.
3) The temporal average volume for the motion-corrected time series was used to generate a brain mask to minimize the inclusion of the extra-cerebral tissues.
4) Spatial normalization to the standard MNI152 template was performed using a 12-parameter affine transformation and mutual-information cost function. During the affine transformation the R-fMRI data were also re-sampled to isotropic resolution using a Gaussian kernel with 4 mm full width at half maximum (FWHM).
5) Nuisance signal removal was performed by voxel-wise regression using 16 regressors based on the motion correction parameters, average signals of the ventricles and white matter as well as their 1^st^ order derivatives.
6) After baseline trend removal up to the 3rd order polynomial,
7) effective band-pass filtering was performed using low-pass filtering at 0.08 Hz.
8) Local Gaussian smoothing up to FWHM = 4mm was performed using an eroded gray matter mask [2].
9) Pearson's correlation coefficients (CC) were then computed between the time courses of all pairs of voxels inside the brain, leading to a whole-brain functional connectivity matrix for each subject. This computation was performed for all voxels located within the brain mask.

The brain mask was generated by overlapping the MNI co-registered brains of all participants and contains 28146 voxels. Each voxel inside the brain was used as the seed voxel in turn. Therefore, the CC matrix size is 28146 × 28146. Each row or column of the CC matrix corresponds to the CC image volume for the seed voxel in question with the rest of the brain and was used to compute a histogram of 200 evenly binned points and derive the different resting-state functional connectivity (RFC) metrics.

### 2.5 Computation of RFC metrics

Based on the histogram for each row of the CC matrix, we derived the following two types of threshold-free voxel-wise RFC metrics: the connectivity strength index (CSI) and connectivity density index (CDI) [3], [4], [5], [6]. As we are interested in investigating systematically all relevant synchronized activities in the whole-brain R-fMRI signals, we quantify the negative and positive portions of the CC histogram separately to avoid information cancelation, which will lead to sensitivity reduction and statistical interference. From here on, the subscripts “N” and “P” are used to denote the negative and positive portions of the RFC metrics, respectively. The metrics without subscripts refer to the mixed measures without distinction of the negative and position contributions.

**Fig. 1:**
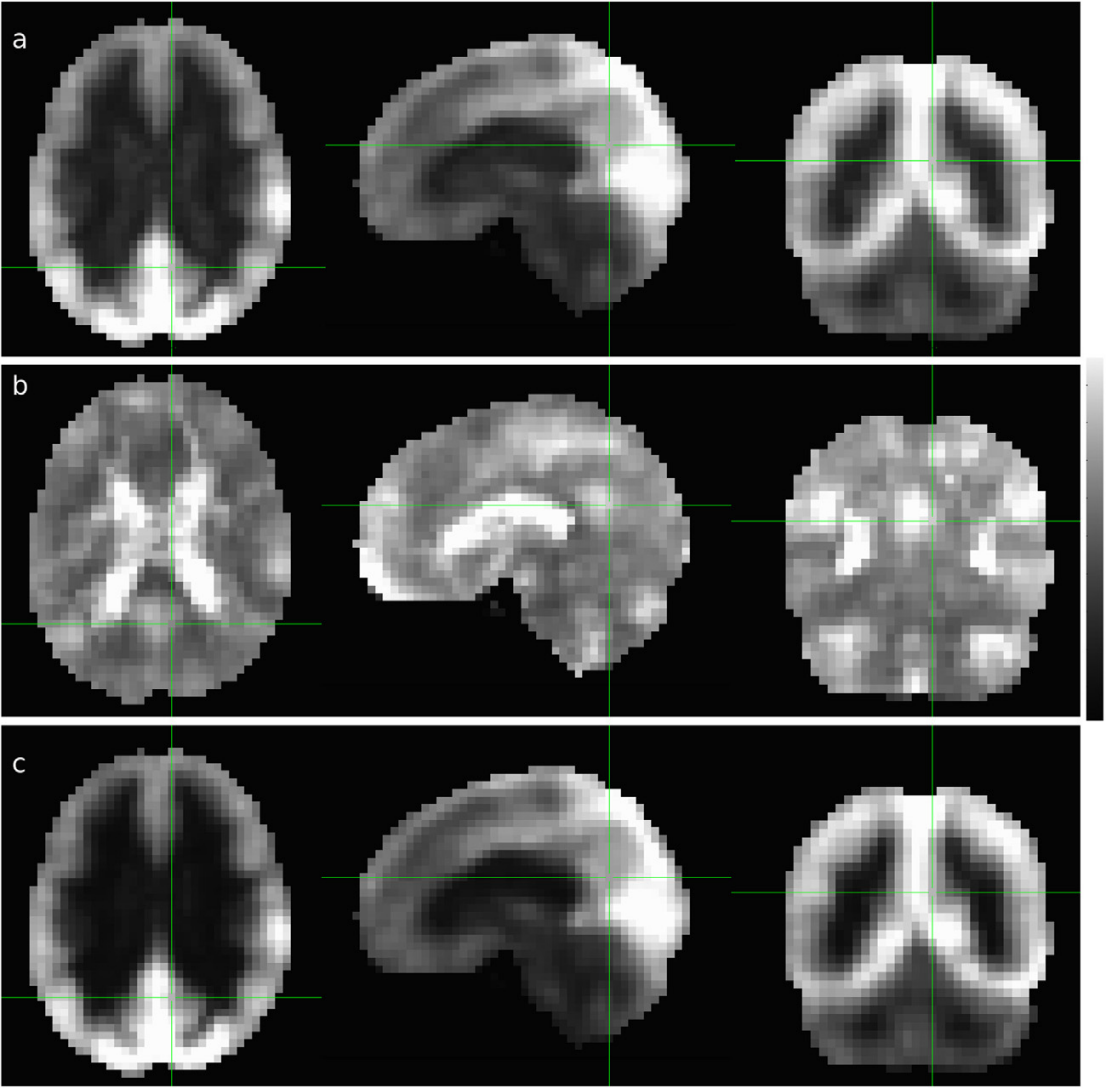
Cross-sectional display of the average CSI (a), CSI_N_ (b) and CSI_P_ (c) for the subgroup of elderly male subjects of 70±6 years old (N=35). The scalar indicates the Z-score levels and the green lines indicate the locations of the cross-sections.

**Fig. 2:**
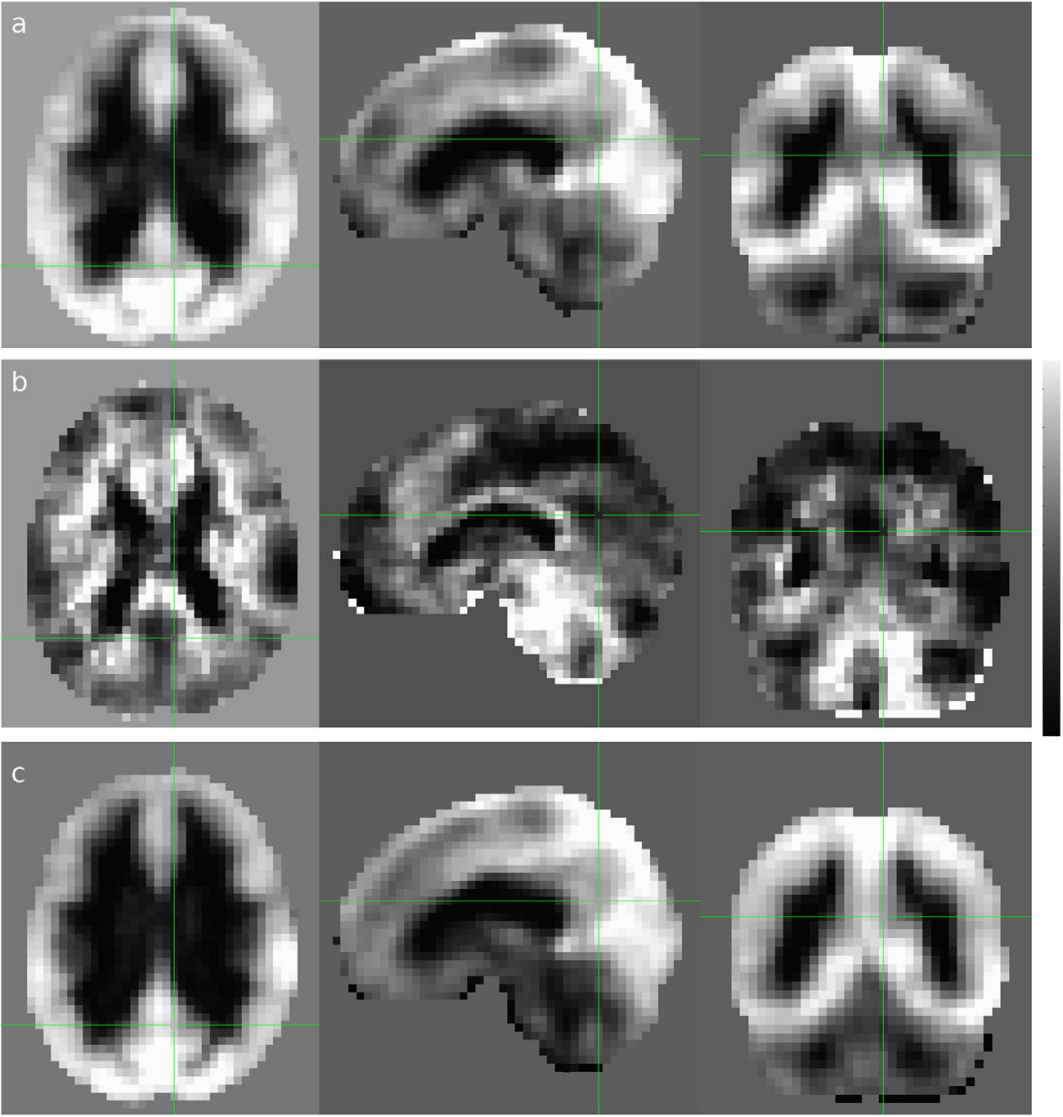
Cross-sectional display of the average CDI (a), CDI_N_ (b) and CDI_P_ (c) for the subgroup of elderly male subjects of 70±6 years old (N=35). The scalar indicates the Z-score levels and the green lines indicate the locations of the cross-sections.

**Fig. 3:**
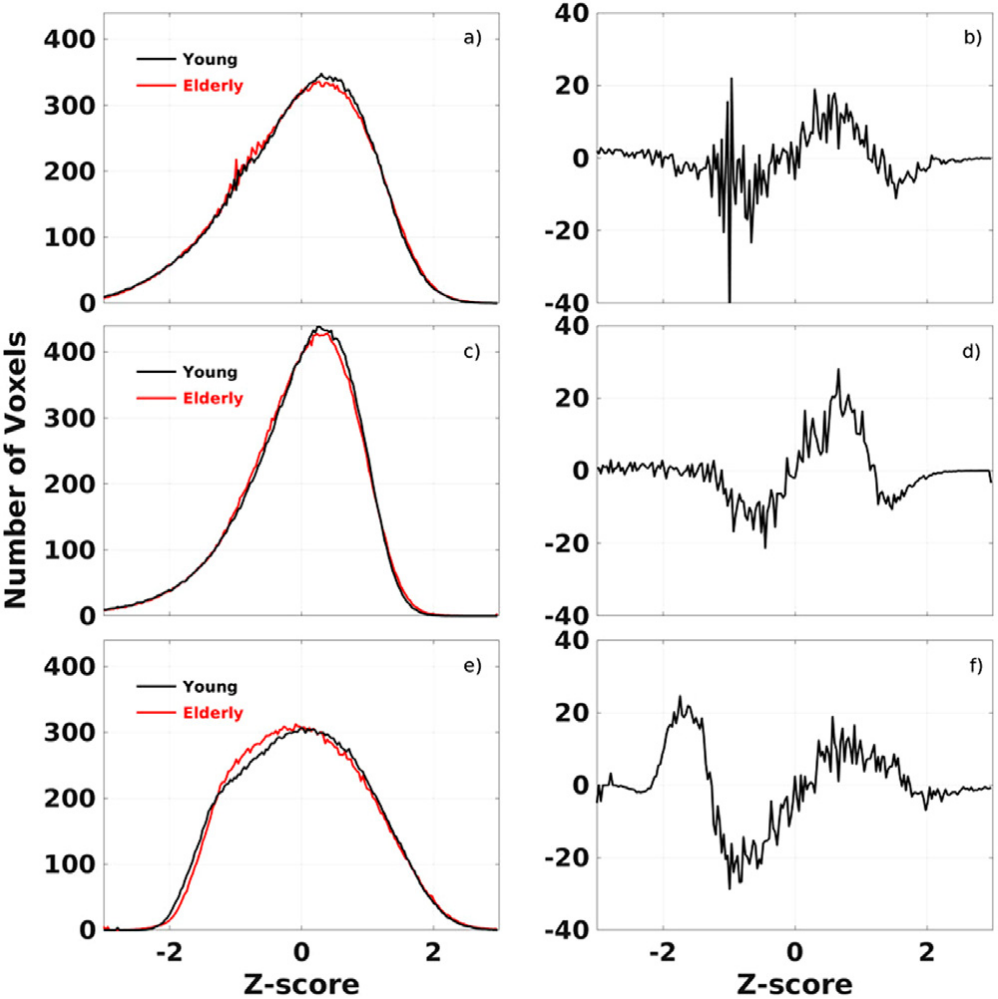
The average histograms of the CSI (a), CSI_P_ (c) and CSI_N_ (e) data for the subgroup of elderly male subjects of 70±6 years old (N=35) and the subgroup of younger male subjects of 25±4 years old (N=47) as well as the corresponding differences between the two subgroups (b, d, and f).

**Fig. 4:**
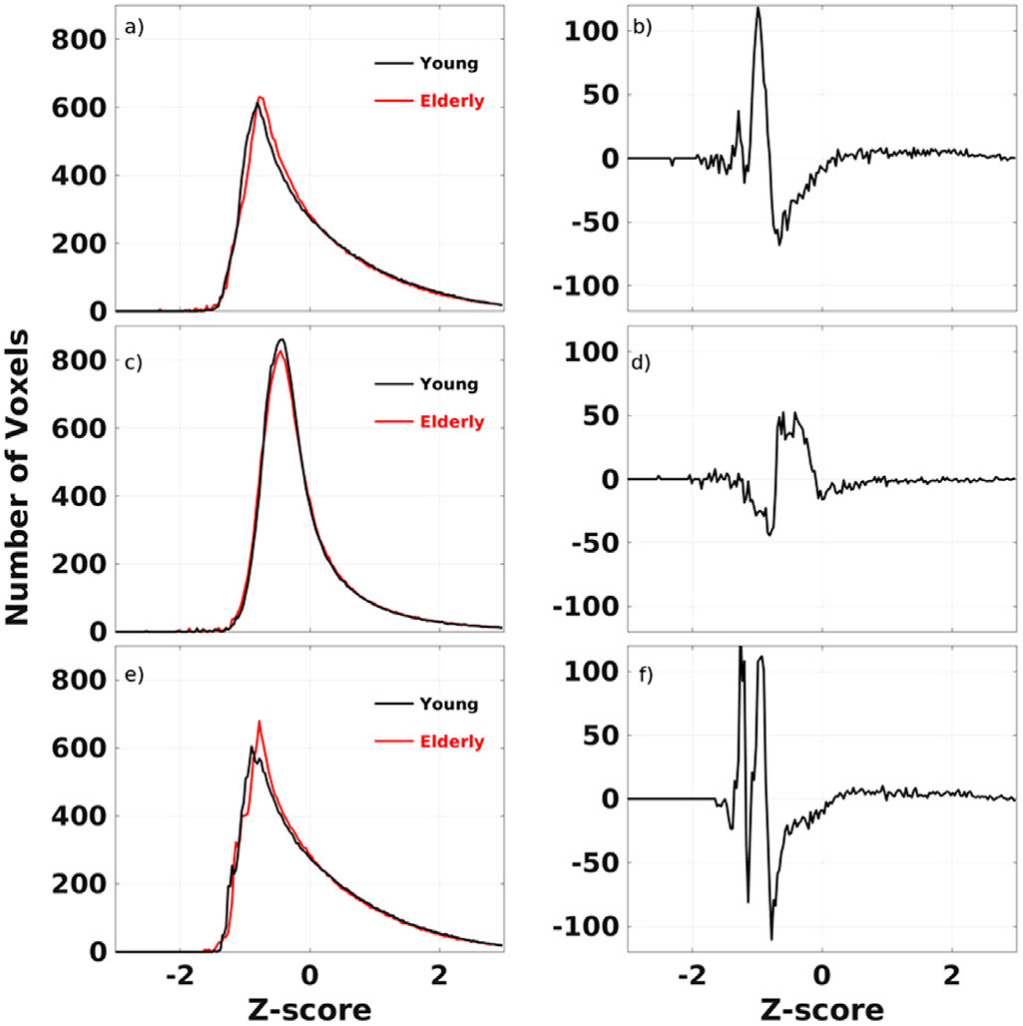
The average histograms of the CDI (a), CDI_P_ (c) and CDI_N_ (e) data for the subgroup of elderly male subjects of 70±6 years old (N=35) and the subgroup of young male subjects of 25±4 years old (N=47) as well as the corresponding differences between the two subgroups (b, d, and f).

The voxel value for the CSI_P_ and CSI_N_ are defined as the averages of the positives and negatives in each row of the CC matrix, respectively. That is

(1) $$\text{CS}I_{P}=\left(\sum_{CC>0}CC_{row}\right)/np$$

(2) $$\text{CS}I_{N}=\left(\sum_{CC<0}CC_{row}\right)/nn$$

Where $CC_{row}$ refers to a row in the CC matrix. *np* and *nn* refer to the number of positive and negative correlation coefficients in a row of the CC matrix, respectively. The voxel values for CDI are defined as the convolution between the CC histogram and a kernel function. That is

(3) $$\text{CDI}\;=\text{Hist}\left(CC_{row}\right)⊗\text{kernel}$$

Similar to the CSI_P_ and CSI_N_, the CDI_P_ and CDI_N_ correspond to the positive and negative portions of the convolution defined in eq. (3), respectively. To facilitate statistical comparison, it is useful to transform these raw RFC metrics into standard Z-score using the following formula:

(4) Z=(RFC−u)/σ

Where μ and σ are the mean and standard dilation of the corresponding RFC metrics, respectively. For optimization of the CDI sensitivity and contrast, we investigated 6 different kernel functions, including

(5) $$k_{i=1,2,\ldots 4}=\left|x^{i}\right|,$$

(6) $$k_{5}=\left|\text{si}n^{2}\left(\pi /2x\right)\right|,$$

(7) $$k_{6}=\text{step}\left(\left|x\right|-0.3\right),$$

Where x⊂ [-1,1] corresponding to the interval of the correlation coefficients. It is obviously that a kernel should weight the higher correlation coefficients more than the lower values. The selection of the kernel is aimed to optimize the contrast and signal-to-noise ratio (SNR) of the derived CDI_P_ and CDI_N_ metrics. The commonly used threshold approach can be considered as the case of the square-well kernel function *k_6_*. For illustration purpose, an arbitrary threshold of 0.3 was used here. The CSI metrics can also be considered as a special case of CDI corresponding to a kernel of the sign function. As discussed above, the derived RFC metrics based on kernel k_4_ are accessible at the following site: http://dx.doi.org/10.17632/3nm3ctwycc.1.

All the RFC metric files are in 4D nifti format with a matrix size=45 × 54 × 45 × 227. As indicated by the matrix size, each volume corresponds to the RFC result for an individual participant and there are 227 volumes corresponding to the 227 subjects of the studied cohort. The 4^th^ dimension of the data is organized according to the alphabet order of the raw data file names. Therefore, it starts with the youngest female subjects and ends with the eldest male participants.

Fig. 1 shows the average CSI, CSI_P_, and CSI_N_ results for the subgroup of all elderly male subjects of 70±6 years old (n=35). The corresponding CDI, CDI_P_, and CDI_N_ results are depicted in Fig. 2. Compared with other RFC metrics documented in the published literature [7], [8], [9], [10], the proposed CSI and CDI metrics can mitigate a number of technical issues such as the arbitrariness in the selection of cut-off threshold, loss of information, and computation difficulty.

The derived RFC metrics can be used for direct statistical comparison to assess differences between subjects, subject groups, and longitudinal changes of individuals. This point is illustrated by the histogram comparisons of the metrics between the elderly and younger male subgroups in the cohort. Figs. 3 and 4, depict the average histogram results for the subgroup of all elderly male subjects of 70±6 years old (n=35) and the subgroup of all younger male subjects of 25±4 years old (n=47), respectively. The positive and negative peaks in the histogram differences indicate that the impact of adult age on the RFC can be either decremental or enhancement. For details regarding to the anatomical locations and functional networks of the involved aging effect can be further analyzed with more rigorous voxel-wise statistical methods.

## Ethics Statement

Participants signed informed consent before completing the MRI examination protocol. They were financially compensated for their participation. The regional ethics committee in Stockholm approved the study protocol 2014/1982-31/1, which was conducted in line with the declaration of Helsinki.

## Declaration of Competing Interest

None.
